# A Cross-Sectional Study of the Dental Arch Relationship and Palatal Morphology after Cleft Surgery in Italian Children with Unilateral Cleft and Lip Palate

**DOI:** 10.3390/children10091559

**Published:** 2023-09-15

**Authors:** Patrizia Defabianis, Rosa Guagnano, Federica Romano

**Affiliations:** 1 Department of Surgical Sciences, C.I.R. Dental School, University of Turin, 10126 Turin, Italy; patrizia.defabianis@unito.it; 2 Private Practice, 10100 Turin, Italy; rosaguagnano@virgilio.it

**Keywords:** orofacial cleft, cleft palate therapy, dental arch, treatment outcome

## Abstract

Dental arch relationships (DARs) and palatal morphology (PM) were evaluated in in non-syndromic complete unilateral cleft lip and palate (UCLP) Italian patients after surgery. Pre- and postnatal factors affecting the results were investigated. Sixty-six children with UCLP (40 boys and 26 girls, with a mean age of 10.1 ± 2.9 years), predominantly Caucasian (77%), were consecutively enrolled in this cross-sectional study. Twenty children had received a one-stage protocol consisting of an early periosteal palate surgical repair and lip closure and forty-six were submitted to a staged surgical protocol with delayed palate repair (DPR). A single clinician collected data on their medical history and carried out a dental examination. The DAR and PM were graded on dental casts according to the Eurocran index and dichotomised as favourable and unfavourable based on the treatment outcome. Multiple logistic regression analyses demonstrated that female sex (OR = 6.08, 95% CI: 1.47–25.23, *p* = 0.013), DPR (OR = 4.77, 95% CI: 1.14–19.93, *p* = 0.032) and the use of a neonatal plate (OR = 4.68, 95% CI: 1.27–17.16, *p* = 0.020) increased the odds of having favourable DAR, while only DPR (OR = 9.76, 95% CI: 2.40–39.71, *p* = 0.001) was significantly associated with a favourable PM. Based on these findings, only DPR had a significantly favourable effect on both DAR and DM in Italian children with complete UCLP.

## 1. Introduction

Cleft lip with or without palate (CL/P) is the most frequent malformation of the craniofacial district, with significant geographic and ethnic variations [1,2]. CLP is less prevalent in Africa (1/2500) and Europe (1/1000) compared to Indo-America and Asia (1/500), while the frequency of CP is similar among ethnic groups (0.5/1000) [2]. In Italy, the prevalence of isolated oro-facial clefts from 2001 to 2014 was estimated to be approximately 1.03 cases per 1000 live births, with unilateral cleft lip and palate (UCLP) affecting one-third of children with a cleft [3].

UCLP involves the face and the oral cavity, impairing aesthetics, eating and speech functions and thereby compromising both children’s and parents’ quality of life [4,5,6,7]. It requires complex management that relies on a multidisciplinary corrective treatment aimed at restoring the cleft defect to the normal range and preserving the normal potential growth of the involved area. The necessary procedures include surgery, orthodontic treatment and laryngologist and speech therapy [8]. Until now, there has been consensus on either the technique or the timing of cleft surgery. an incomplete understanding of the individual factors contributing to the treatment outcome has led to a great diversity in surgical procedures [9]. Staged protocols have been introduced to limit the restraining effect of palatoplasty on maxillary growth by postponing surgery to times with less impact on craniofacial development [10,11,12]. Conversely, one-stage methods, in which lip and palate repair are performed in a single session, have been demonstrated to improve speech and feeding function, even if their effect on mid-facial growth is less clear [13,14,15]. The contributing roles of other potential pre-natal factors (such as sex, age, ethnicity, the size of the cleft and the side affected) on treatment outcomes have also been addressed in the literature with conflicting results [16,17,18]. Meazzini et al. identified agenesis of lateral incisors as one of the most relevant factors predisposing cleft patients to maxillary hypoplasia [19].

The Eurocran index (EI) was introduced to assess treatment outcomes following cleft surgery [20]. This index, when compared to other extensively used grading systems, like the GOSLON Yardstick for late mixed/early permanent dentition and the 5-year-olds’ (5YO) index for deciduous dentition [21,22], has the advantage of rating not only the occlusal relationship in all three planes of space (including te displacement of the lesser segment on the cleft side) but also the palatal morphology (see Table 1). This is a relevant issue considering the negative effect due to wound contraction and tissue scarring on maxillary growth [23]. The EI has been found to be an accurate and reliable tool for grading the quality of treatment on both plaster and digital dental models of ULCP children [14,20,24]. However, information about factors influencing treatment outcomes is still controversial and limited to Poland, Switzerland, and some Asian countries [14,17,18,25], while no data are available from Italy.

Therefore, the aim of the present study is to examine the dental arch relationship and palatal morphology in non-syndromic Italian children with complete UCLP after cleft surgery, using the EI, and to explore the pre-natal/congenital and post-natal/environmental factors that may influence them. The null hypothesis is that such factors have no impact on treatment outcomes.

## 2. Materials and Methods

This single-centre cross-sectional study was carried out in accordance with the Declaration of Helsinki and was reported according to the STROBE guidelines. Ethical approval was obtained from the Institutional Ethics Committee (No. 0038526). Children were enrolled in the study after their parents/guardians provided written informed consent.

### 2.1. Study Setting and Population

Children with a clinical diagnosis of non-syndromic complete ULCP were consecutively recruited among those referred from the Plastic Surgery Division of the Regina Margherita Children Hospital of Turin to the Section of Pediatric Dentistry, C.I.R. Dental School, University of Turin, for clinical examination from July 2019 to November 2022. Only children of both sexes, aged up to 16 years, who resided in Italy, irrespective of their provenience, and who were previously treated with cheiloplasty and palatoplasty were included in this study. Children with other cleft phenotypes or associated syndromes or those who were previously treated with alveolar bone graft or orthodontics were excluded.

### 2.2. Data Collection

The data extracted from the patients’ medical records were age, sex, race/ethnicity, side of UCLP, family history of UCLP, type and timing of orthopaedic treatment and surgical repair techniques. Race/ethnicity was confirmed via an interview with the parents of each child and classified into four categories: white (Caucasian), Black, Hispanic and Asian.

Children were classified according to the time at which surgical protocols were performed: those who received one-stage palatoplasty in which both the hard and soft palates were closed at the same time were classified as early palate surgical repair (EPR) patients, while those who underwent a staged protocol were classified as delayed palate surgical repair (DPR) patients. Each enrolled patient was examined by a single trained specialist in paediatric dentistry, and intraoral photographs and dental impressions were also taken. Orthopantomography of the dental arches was performed for all participants, and the absence of upper permanent lateral incisors was assessed through clinical and radiographic evaluation.

Another examiner, who was not involved in the clinical examination, blindly scored all the dental casts using the EI [20]. Both components of the EI were graded: (1) dental arch relationship (DAR), scored from 1 to 4 in which 1 corresponds to a good treatment outcome and 4 corresponds to a poor outcome and the need for orthognathic surgery, and (2) palatal morphology (PM), scored from 1 to 3 in which 1 indicates good morphology and 3 indicates poor morphology. To ensure the reliability of the scoring method, the examiner received initial training with a clinician experienced in the treatment of cleft patients. Afterwards, they graded 20 dental models which were not included into the study sample twice, with an interval of two weeks between the first and second sessions. The intra- and inter-rater agreements were analysed using kappa statistics, obtaining Cohen’s kappa values ≥0.88 and ≥0.85, respectively, representing very good agreement according to Altman et al. [26].

### 2.3. Statistical Analysis

The sample size was calculated based on the percentage of successful DARs reported in Western European UCLP children [25], with a precision of 10% and a level of significance of 5%. The minimum number of children to enrol into this study was 60. For the regression analysis, considering five potential predictors (sex, race/ethnicity, the timing of the surgical closure of the lip and palate cleft, agenesis of lateral incisor and previous orthopaedic treatment) and a ratio of 1 predictor to 12 cases [18], the required number of participants was 60.

Continuous variables were summarised as means ± standard deviation, and qualitative variables were summarised as absolute and relative frequencies. DARs and PMs were dichotomised as favourable (ratings 1 and 2 for the DAR and rating 1 for the DM) and unfavourable categories (ratings 3 and 4 for the DAR, and ratings 2 and 3 for the PM) based on the treatment outcomes [27]. The chi-squared test or Fisher’s exact test was used to evaluate which study variables were associated with a favourable DAR and DM.

A binary logistic regression analysis was performed using the dichotomous DAR and PM as dependent variables. Both univariate and backward stepwise multiple logistic regression analyses were performed to explore congenital and environmental factors affecting the DAR and PM in UCLP patients. Associations were reported as odds ratio (ORs) and 95% confidence intervals (CIs).

The data were analysed using SPSS software, version 28.0 (IBM Corp, Armonk, NY, USA). Two-tailed *p* values less than 0.05 were considered statistically significant.

## 3. Results

### 3.1. Study Population

Sixty-six UCLP participants (40 boys and 26 girls), aged from 6 to 16 years (with a mean age of 10.1 ± 2.9 years), were included in the study, with 51 Caucasian (77.3%), 8 Asian (12.1%), 3 Hispanic (4.5%), and 4 Black (6.1%) children. Forty-two patients (63.6%) were affected by UCLP on the left side, and twenty-four (36.4%) patients were affected on the right side. None of them presented Simonart’s bands. One child had a family history of cleft.

Forty-six children (with a mean age of 10.8 ± 2.8 years) were treated according to the DPR protocol for cleft repair from January 2005 to December 2016 at the Plastic Surgery Division of the Regina Margherita Children Hospital of Turin. They received cheiloplasty (via modified Millard or Noordhoff techniques) at an average age of 6.1 ± 2.4 months and soft palate repair at 13.2 ± 3.3 months (utilizing the Widmaier–Perko method combined with V-Y soft palate repositioning). The hard palate was closed at 4.4 ± 0.9 years according to the Schweckendiek technique with a mucoperiosteal flap.

Twenty patients (with a mean age of 8.5 ± 2.4 years) underwent an EPR protocol in other cleft centres in North Italy between January 2009 and December 2015. A one-stage periosteal palatoplasty and cheiloplasty were carried out at a mean age of 6.8 ± 6.1 months. For lip repair, either a modified Tennison–Randall method or modified Mulliken method was used, while for palatoplasty, either the Bardach technique or Von Langenbeck technique was applied.

Infant orthopaedic treatment was carried out in 38 children using a pre-surgical nasoalveolar moulding (PNAM) procedure.

### 3.2. Analysis of Dental Arch Relationship and Palatal Morphology

Table 2 reports the distribution of DAR and PM scores and the status of the maxillary lateral incisors for the overall sample and according to the surgical protocols. An unfavourable dental arch relationship (DAR scores of 3 and 4) was observed in 25/66 (37.9%) of the UCLP children, and moderate-to-severe changes in palatal morphology (PM scores of 2 and 3) were observed in 27/66 (40.9%) of them. The lateral permanent incisor was missing on the cleft-side in 22/66 (33.3%) patients and in only 9/66 (13.6%) on the non-cleft side. Bilateral agenesis was detected in 3/66 (4.5%) children. More than 76% of the children treated with DPR had DAR scores of 1 and 2 and a PM score of 1 compared to 30.0% and 20%, respectively, of those treated via EFP.

As reported in Table 3, the DAR and PM treatment outcomes were significantly associated with race/ethnicity (*p* = 0.003 and *p* = 0.005), the type of surgical protocol (*p* = 0.001 and *p* < 0.001), and the use of a neonatal maxillary plate (*p* < 0.001 and *p* = 0.021). Sex (*p* = 0.004) showed a statistically significant association only with the DAR, whereas agenesis of the non-cleft-side lateral incisor was only associated with PM (*p* = 0.040). Age and the side of the cleft were not associated with the treatment outcomes.

Table 4 and Table 5 show the crude and adjusted odd ratios for factors affecting DAR outcomes. An unadjusted logistic regression analysis revealed that female sex, DPR protocol, and the use of a neonatal maxillary plate were associated with increased odds of having a favourable treatment outcome, which was found to be statistically significant (Table 4). The association with Caucasian ethnicity was on the borderline of statistical significance (*p* = 0.048).

When entered into the multiple logistic regression model, the ORs of all the variables apart from Caucasian ethnicity remained statistically significantly associated with a favourable DAR, but with a wide CI. The adjusted OR for the DPR and infant orthopaedics decreased with respect to the crude value (Table 5).

Table 6 reports the crude ORs for factors associated with PM treatment outcomes. The DPR protocol and the use of neonatal maxillary plate were associated with higher odds of producing a favourable PM, while agenesis of lateral incisor on the non-cleft side was associated with an unfavourable treatment outcome. In the multiple regression model, the DPR surgical protocol was the only variable that remained statistically significantly associated with a favourable PM, with the adjusted OR decreasing compared to the crude value (adjusted OR = 9.76, 95% CI: 2.40–39.71, *p* = 0.001).

## 4. Discussion

Although all surgical techniques of oro-facial cleft repair have been shown to negatively impact maxillary bone development, it is not possible to draw any definitive conclusion due to the heterogeneity of the studies available in the literature regarding the experimental design, the duration of follow-up, the timing and type of surgery and the indices used to evaluate the treatment results [15,28].

In the current study we explored the influence of pre-natal/phenotype (race/ethnicity, sex, cleft side and family history) and post-natal/environmental factors (pre-surgical orthopaedic treatment and the timing of surgical protocols) on the treatment outcomes in Italian children with UCLP using the EI. The EI rates the palatal morphology (PM) and the degree of malocclusion in the sagittal, transversal and coronal planes (DAR), including the displacement of the lesser segment on the cleft side [14,29]. Furthermore, it provides a more detailed guideline for the categorization of surgical outcomes with respect to the GOSLON Yardstick grading system, which has been the most commonly used index thus far in the literature [14]. The EI scores were categorised into favourable and unfavourable outcomes, and the children were divided accordingly. The children included in the favourable group are those who are more likely to receive conventional orthodontics, while those included in the unfavourable group may need later surgical correction [27]. Furthermore, EI has never been employed in the Italian population, even if some studies questioned the reliability of the PM component due to the poor inter-rater agreement [24,29].

To our knowledge, only five studies had evaluated the effect of surgical treatment on maxillary growth using this index [14,17,18,20,25]. Fudalej et al. [20] compared DAR and PM in preadolescent Polish children with UCLP treated with a one-stage surgical protocol differing only in the palatoplasty technique employed. A worse DAR was obtained in children in which the palatal bone was left denuded on the non-cleft side, inducing scar formation compared to those who received a repair via vomerplasty; the PM in both groups was comparable. In another study, Fudalej et al. [14] reported that the use of one-stage palatoplasty resulted in a poorer DAR but a more favourable PM compared to three-stage surgical repair. Haque et al. [18] demonstrated that the complete UCLP phenotype and the modified Millard technique for cheiloplasty significantly impaired both the DAR and PM in Bangladeshi children. Yew et al. [17] did not find any statistically significant association between different cheiloplasty and palatoplasty techniques and the DAR in Malay children with UCLP. Finally, Benitez et al. [25] reported that the DAR and PM were considerably compromised following one-stage UCLP repair in Swiss children, regardless of primary alveolar bone grafting.

We obtained percentages of favourable DARs and PMs similar to those reported in the Swiss and Polish studies [14,20,25] using an EPR protocol but better than those achieved by Haque et al. with delayed surgical protocols [18]. Different surgical timings and the surgical techniques used could account for these discrepancies.

In the present study, univariate logistic regression analyses demonstrated that race/ethnicity, sex, the timing of hard palate closure, the use of infant orthopaedics and agenesis of non-cleft lateral incisor were significantly associated with surgical treatment outcomes. Interestingly, Caucasian children with UCLP showed better DAR and PM scores compared to the other ethnic groups, while the Asian and Hispanic patients received the worst results. The impact of craniofacial morphology should not be under-evaluated as a high frequency of skeletal class III malocclusion has been reported in the Asian population, which is characterised by a small and flat median nasal process and a small middle third of the face [17,30]. However, such associations were no longer statistically significant in the multiple logistic models, probably due to the minority of non-Caucasian children.

In line with previous studies, we observed that UCLP was more prevalent in males than females and more common on the left side [16,17,31]. Females also exhibited better DARs than males, whereas the cleft side did not show any influence. Gender-specific differences in the treatment outcomes after cleft surgery have been seldom addressed in the literature. The majority of available studies referred to Asian populations and reported conflicting results. Yew et al. [17] did not find any association between sex and the DAR outcome in Malay UCLP subjects, while Haque et al. [32] reported more favourable DARs among Bangladeshi females with UCLP. Considering the multitude of elements influencing the treatment of UCLP, it is difficult to explain the reasons for such findings. Aside from the impact of genetics, differences in timing and the mechanisms underlying craniofacial growth could be envisaged [33,34]. Orofacial clefts in females mainly occur in the late embryonic period, are mostly clefts of the secondary palate and are usually the result of a fusion defect. Clefts in males more commonly occur in both the early and late embryonic periods, are therefore more often combined clefts of the primary and secondary palates and are more frequently the result of differentiation defects and combined fusion and differentiation defects [34]. The role of hormonal factors should be also taken into account. In particular, oestrogens promote pre- and post-natal growth and the development of craniofacial structures as they regulate bone formation and homeostasis in both sexes. Female children experience bone maturation earlier than male children, probably under the influence of the higher levels of oestrogens. The role of oestrogens in the development of the dental arches was demonstrated in an animal model [35]. This gendered effect on treatment outcomes warrants further investigation, mainly in children with UCLP from Western countries.

The DPR protocol, which postponed hard palate closure until 4–5 years of age, was associated with more favourable DAR and PM outcomes. A recent meta-analysis reported better DARs when repairing the hard palate after the age of three [36]. Consistent with these findings, Hak et al. stated that the detrimental effects of cheiloplasty and palatoplasty on anterior arch growth lasted until the age of 5 [37]. A recent longitudinal study reported similar favourable DAR scores using the GOSLON Yardstick index when comparing Swedish children treated with a two-stage protocol at 3 and 8 years of age [38]. However, the children operated on at the age of 3 showed better speech development.

According to Liao et al., it seems that it is the timing of hard palate closure and not the sequence of hard or soft palate repair that impacts postoperative growth [39]. Indeed, Mommaerts et al. [40] and Richard et al. [41] found no differences in post-surgery maxillary growth between one-stage and two-stage protocols when the hard palate repair was performed at the same time. Previous studies also claimed no maxillary growth benefit of one procedure of hard palate closure over another [17]. However, contrasting results have been reported in the literature. A recent randomised controlled study did not find any statistically significant difference in DAR outcomes between EPR with the hard and soft palates repaired at 3–4 months and DPR in which the hard palate closure was postponed to 12 or 36 months. No data were reported for the PM because the authors did not find the parameter to be acceptably reliable [42]. Stein et al. observed a similar palatal height but a smaller transverse distance in the upper anterior arch following EPR which tended to compensate at the ages of 15 to 18 years, reaching values comparable to DPR [43].

Considering that there is no definitive evidence as to what the ideal time for palate closure is, the advantages of delayed protocols versus the early one-stage closure of both the hard and soft palates should be carefully evaluated. Malocclusion is a common problem in cleft patients and is basically due to an underdeveloped, hypoplastic maxilla; as consequence, cross-bite and open bite are the malocclusions most often detected, especially on the cleft side [44]. Furthermore, we cannot forget the negative effects on maxillary growth due to wound contraction and tissue scarring [23]. The lack of a palatal suture acting as growth factor and the presence of a scar on the palate result in the mesial rotation of the palatal bone, especially in the frontal region. This will have a negative impact on the development of the maxilla, whose stability in adulthood will be obtained via maxillary segmentalisation after orthognathic surgery. It is also important to evaluate the vertical and sagittal relations of the jaws for a three-dimensional assessment of the malocclusion [45]. The impact of the surgical protocol on masticatory and speech functions should also not be neglected. Feeding improvement and better phonetic development have been related to EPR [25]; however, these functional issues were not addressed in the present study.

Furthermore, we found pre-surgical orthopaedic treatment to be associated with better DAR and PM scores. The use of a neonatal maxillary plate optimises the therapeutic results as it improves tongue function and allows for growth guidance of the palatal segments, facilitating the surgical correction [46,47,48]. In the present study, a pre-surgical PNAM was used to change the maxillary width in the canine and molar region as well as the arch form in order to make the surgical union easier while improving nasal morphology [49,50]. The pre-surgical preparation may also require a NAM-plate, an acrylic orthodontic device aimed at rotating the premaxilla, thus reducing the amount of cleft fissure. It should be applied as soon as possible and worn daily [51]. In this way, maxillary growth will be stimulated in order to change the growth pattern in patients with an oro-facial cleft [44,52]. However, the long-term benefits of these appliances are still debated [53]. Mishima et al. reported the influence of palatal plates on 3D maxillary development occurring in the first 4 months of life [54].

In contrast with previous reports, none of the UCLP patients enrolled in this study presented Simonart’s bands, which may have a favourable effect on the oro-facial outcome, making lip and palate repair procedures less traumatic [55]. Indeed, they affect the morphology of the maxillary dental arch and direct the anterior end of the non-cleft segment closer to the cleft segment. Their presence would seem also to be associated with less hypoplastic embryological processes in the maxilla, leading to a lower prevalence of lateral incisor agenesis distally to the cleft area [56].

Meazzini et al. identified agenesis of lateral incisors as one of the most relevant factors predisposing cleft patients to maxillary hypoplasia [19]. Surprisingly, we observed that agenesis of the non-cleft lateral incisor reduced the odds of achieving favourable treatment outcomes in terms of the PM but not the DAR, even if the association approached statistical significance. Missing lateral incisors were found to predispose UCLP children to maxillary hypoplasia and to increase the need for orthodontic treatment and orthognathic surgery [19,57,58]; the main problem is the maxillary asymmetry favouring the development of cross-bite.

It is of note that in the multiple regression models, DPR remained significantly associated with both the DAR and PM, whereas sex and infant orthopaedics were only associated with the DAR. This may emphasise the relevant role of the timing of the palate closure on treatment outcomes in spite of the wide interval confidence of the association.

These data should be interpreted within the limitations of the current cross-sectional study that enrolled UCLP patients without Simonart’s bands, who were mostly of Caucasian ethnicity, in a university-based Italian dental centre. This may limit the external generalizability of the present results. Furthermore, the sample size was small, even if it was consistent with the number of children enrolled in previous studies due to the rarity of a complete UCLP phenotype, and this could have resulted in a reduction in the statistical power of the study. Information on the initial size of the defect was not available even if cleft size might influence the treatment outcome.

Furthermore, the inability to assess the impact of genotype on craniofacial growth and having multiple operators performing different surgeries may have influenced the treatment outcomes. Finally, patient-related outcomes, in terms of aesthetic appearance, feeding and speech development, were not recorded. Thus, further multi-centre studies with larger numbers of children of different races/ethnicities are needed to help clinicians adopt the most appropriate approach to optimising both maxillary bone development and speech outcomes in affected patients.

## 5. Conclusions

The better surgical protocol for complete UCLP is still a subject of controversy. Based on the present results, DPR is associated with more favourable treatment outcomes in terms of both the DAR and PM compared to EPR. Female sex and the use of pre-surgical orthopaedics would seem to improve only DAR outcomes in Italian UCLP children.

## Figures and Tables

**Table 1:** Grading according to the Eurocran Index (modified by Fudalej et al. 2011 [14]).

Grades	Dental Arch Relationship (DAR)
1	Class I or Class II apical base relationship. Positive overjet and overbite, or markedly increased overjet but no overbite at both central incisors. Case classified as grade 2 in absence of overbite and considerably increased overjet.
2	Class I apical base relationship. Positive overjet and overbite at the non-cleft incisor; stable overjet and overbite at the cleft-side incisor following tilting or derotation. Case classified as grade 3 in the case of moderate open bite.
3	Edge-to-edge or mild skeletal class III apical base relationship. One or both central incisors are edge-to-edge or in a close anterior cross-bite.
4	(a) Class III apical base relationship. Anterior cross-bite at both central incisors or anterior cross-bite at one incisor and edge-to-edge for the other one. (b) As grade 3 but with marked open bite.
**Grades**	**Palatal Morphology (PM)**
1	Good anterior and posterior height; minor surface irregularities; nil or minor deviation of the arch form.
2	Moderate reduction in anterior and posterior height; moderate surface irregularities; moderate deviation of the arch form.
3	Severe reduction in palate height; severe surface irregularities; severe deviation in the arch form.
	The initial grade is based on the worst feature of the three and it may be modified depending on the degree of severity of the other ones.

**Table 2:** EUROCRAN Index and agenesis of lateral permanent incisors in the overall sample and according to the surgical protocols.

Variables [n (%)]	UCLP Children (n = 66)	DPR (n = 46)	EPR (n = 20)
Eurocran dental arch relationship scores			
1	11 (16.7)	11 (23.9)	0 (0.0)
2	30 (45.5)	24 (52.2)	6 (30.0)
3	14 (21.2)	8 (17.4)	6 (30.0)
4	11 (16.7)	3 (6.5)	8 (40.0)
Eurocran palatal morphology scores			
1	39 (59.1)	35 (76.1)	4 (20.0)
2	22 (33.3)	10 (21.7)	12 (60.0)
3	5 (7.6)	1 (2.2)	4 (20.0)
Missing upper lateral incisor—cleft side			
Yes	22 (33.3)	11 (23.9)	11 (55.0)
No	44 (66.7)	35 (76.1)	9 (45.0)
Missing upper lateral incisor—non-cleft side			
Yes	9 (13.6)	3 (6.5)	6 (30.0)
No	57 (86.4)	43 (93.5)	14 (70.0)
Missing upper lateral incisor—bilateral			
Yes	3 (4.5)	1 (2.2)	2 (10.0)
No	63 (95.5)	45 (97.8)	18 (90.0)

DPR: delayed palate repair surgery; EPR: early palate repair surgery.

**Table 3:** Demographic and surgical variables according to the dental arch relationship (DAR) and palatal morphology (PM) treatment outcomes.

Variables	DAR			PM		
	Favourable	Unfavourable	*p* Value	Favourable	Unfavourable	*p* Value
Sex (n (%))			0.004			0.062
Male	19 (47.5)	21 (52.5)		20 (50.0)	20 (50.0)	
Female	22 (84.6)	4 (15.4)		19 (73.1)	7 (26.9)	
Age (years, mean ± SD)	10.4 ± 2.8	9.6 ± 2.9	0.273	10.3 ± 2.5	9.7 ± 3.3	0.406
Race/Ethnicity (n (%))			0.003			0.005
Caucasian	35 (68.6)	16 (31.4)		33 (64.7)	18 (35.5)	
Asian	2 (25.0)	6 (75.0)		2 (25.0)	6 (75.0)	
Hispanic	0 (0.0)	3 (100.0)		0 (0.0)	3 (100.0)	
Black	4 (100.0)	0 (0.0)		4 (100.0)	0 (0.0)	
Cleft side (n (%))			0.962			0.539
Left	26 (61.9)	16 (38.1)		26 (61.9)	16 (38.1)	
Right	15 (62.5)	9 (37.5)		13 (54.2)	11 (45.8)	
Missing lateral incisor – Cleft side (n (%))			0.370			0.595
Yes	12 (54.5)	10 (45.5)		12 (54.5)	10 (45.5)	
No	29 (65.9)	15 (34.1)		27 (61.4)	17 (38.6)	
Missing lateral incisor – Non-cleft side (n (%))			0.072			0.040
Yes	3 (33.3)	6 (66.7)		2 (22.2)	7 (77.8)	
No	38 (66.7)	19 (33.3)		37 (64.9)	20 (35.1)	
Surgical Protocol (n (%))			0.001			<0.001
DPR	35 (76.1)	11 (23.9)		35 (76.1)	11 (23.9)	
EPR	6 (30.0)	14 (70.0)		4 (20.0)	16 (80.0)	
Neonatal maxillary plate (n (%))			<0.001			0.021
Yes	31 (81.6)	7 (18.4)		27 (71.1)	11 (28.9)	
No	10 (35.7)	18 (64.3)		12 (57.1)	16 (42.9)	

DPR: delayed palate repair surgery; EPR: early palate repair surgery; SD: standard deviation.

**Table 4:** Univariate logistic regression analysis: favourable vs. unfavourable dental arch relationships (DARs).

Variables	Crude Effect		
	Odds Ratio	95% Confidence Interval	*p* Value
Sex			0.004
Male	1.00		
Female	6.08	(1.77, 20.86)	
Age (years)	1.11	(0.92, 1.33)	0.270
Ethnicity			0.048
Non-Caucasian	1.00		
Caucasian	3.28	(1.02, 10.79)	
Missing non-cleft-side lateral incisor			0.068
No	1.00		
Yes	0.25	(0.56, 1.11)	
Surgical Protocol			0.001
EPR	1.00		
DPR	7.42	(2.30, 23.97)	
Neonatal maxillary plate			<0.001
No	1.00		
Yes	7.97	(2.58, 24.60)	

DPR: delayed palate repair surgery; EPR: early palate repair surgery.

**Table 5:** Multiple logistic regression analyses (adjusted odds ratio; backward method): favourable vs. unfavourable dental arch relationships (DARs).

Variables	Adjusted Effect		
	Odds Ratio	95% Confidence Interval	*p* Value
Sex			0.013
Male	1.00		
Female	6.08	(1.47, 25.23)	
Surgical Protocol			0.032
EPR	1.00		
DPR	4.77	(1.14, 19.93)	
Neonatal maxillary plate			0.020
No	1.00		
Yes	4.68	(1.27, 17.16)	

DPR: delayed palate repair surgery; EPR: early palate repair surgery.

**Table 6:** Univariate logistic regression analysis: favourable vs. unfavourable palatal morphology (PM).

Variables	Crude Effect		
	Odds Ratio	95% Confidence Interval	*p* Value
Sex			0.066
Male	1.00		
Female	2.71	(0.94, 7.88)	
Age (years)	1.08	(0.90, 1.29)	0.400
Ethnicity			0.093
Non-Caucasian	1.00		
Caucasian	2.75	(0.84, 8.97)	
Missing non-cleft side lateral incisor			0.028
No	1.00		
Yes	0.15	(0.03, 0.82)	
Surgical Protocol			<0.001
EPR	1.00		
DPR	12.72	(3.51, 46.15)	
Neonatal maxillary plate			0.023
No	1.00		
Yes	3.27	(1.17, 9.13)	

DPR: delayed palate repair surgery; EPR: early palate repair surgery.

## Data Availability

The dataset used and analysed during the current study is available from the corresponding author upon reasonable request.
